# Characterisation, procedures and heritability of acute dietary intake in the Twins UK cohort: an observational study

**DOI:** 10.1186/s12937-022-00763-3

**Published:** 2022-02-27

**Authors:** Emily R. Leeming, Olatz Mompeo, Pauline Turk, Ruth C. E. Bowyer, Panayiotis Louca, Abigail J. Johnson, Tim D. Spector, Caroline Le Roy, Rachel Gibson

**Affiliations:** 1 Department of Twin Research, King’s College London, London, SE1 7EH UK; 2 AgroParisTech, 75231 Paris Cedex 05, France; 3 Division of Epidemiology and Community Health, University of Minnesota, Minneapolis, MN 55454 USA; 4 Department of Nutritional Sciences, King’s College London, London, SE1 9NH UK

**Keywords:** Dietary intake, Food record, Heritability, Diet diary, Eating behaviours, Food frequency questionnaires

## Abstract

**Background:**

Estimated food records (EFR) are a common dietary assessment method. This investigation aimed to; (1) define the reporting quality of the EFR, (2) characterise acute dietary intake and eating behaviours, (3) describe diet heritability.

**Methods:**

A total of 1974 one-day EFR were collected from 1858 participants in the TwinsUK cohort between 2012 and 2017. EFR were assessed using a six-point scoring system to determine reporting quality. The frequency and co-occurrence of food items was examined using word clouds and co-occurrence networks. The impact of eating behaviours on weight, BMI and nutrient intake were explored using mixed-effect linear regression models. Finally, diet heritability was estimated using ACE modelling.

**Results:**

We observed that 75% of EFR are of acceptable reporting quality (score > 5). Black tea and semi-skimmed milk were the most consumed items, on an individual basis (respectively 8.27, 6.25%) and paired (0.21%) as co-occurring items. Breakfast consumption had a significantly (*p* = 5.99 × 10^− 7^) greater impact on energy (kcal) (mean 1874.67 (±SD 532.42)) than skipping breakfast (1700.45 (±SD 620.98)), however only length of eating window was significantly associated with body weight (kg) (effect size 0.21 (±SD 0.10), *p* = 0.05) and BMI (effect size 0.08 (±SD 0.04), *p* = 0.04) after adjustment for relevant covariates. Lastly, we reported that both length of eating window (h2 = 33%, CI 0.24; 0.41), and breakfast consumption (h2 = 11%, CI 0.02; 0.21) were weakly heritable.

**Conclusions:**

EFR describing acute dietary intake allow for eating behaviour characterisation and can supplement habitual diet intake assessments. Novel findings of heritability warrant further investigation.

**Supplementary Information:**

The online version contains supplementary material available at 10.1186/s12937-022-00763-3.

## Background

The human diet is complex and highly variable with an extensive impact on human health [1]. Explorations into diet-disease relations have predominantly assessed the impact of habitual nutrient intake and dietary patterns. However, characterisation of the effects of acute dietary intake, defined as food consumed within a 24-h (or multiple) period(s), provides a detailed short-term snapshot of food and nutrient intake vs. habitual dietary intake measures which provide an average profile over a longer period such as the past month or year. This may assist in disentangling the complex interactions of metabolic biomarkers and gut microbial signatures [2]. Several methods have been developed to collect diverse aspects of dietary data [3], such as estimated food records (EFR) and 24-h food recalls which measure acute dietary intake. The primary difference between EFRs and 24-h recalls is that with 24-h recalls the dietary data is collected through an open-ended interview typically by a trained dietitian, with a key feature being that more detailed information requested from the respondent where appropriate than first reported [4, 5]. EFR are a prospective, self-administered method that aim to capture a participant’s short-term food and drink intake over one or more 24-h period [3, 6]. Traditionally participants receive a paper-based recording form with written instructions [7]. Portion sizes are estimated by the participant, however, weight or volume measures, and visual aids such as photographs, may be used [3, 7]. Depending on the research requirements, recording of further information may be requested. These may include details of the time of consumption, eating occasion, eating location, brand information, and cooking and preparation methods [3, 6]. In this manner, EFR in comparison to habitual diet measures such as Food Frequency Questionnaires (FFQs) allow for the contextual evaluation of meal and snack patterns and other eating habits such as the impact of location (e.g. eating in front of TV vs. communal setting) [7], alongside capturing the complexity of an individual’s food and nutrient intake. EFRs have previously been used to characterise for example portion sizes by the level of income as part of the National Diet and Nutrition Survey (2008–11) [8] to investigating the impact of diet on metabolic syndrome [9].

Food items are predominantly consumed in combination with others, allowing for the characterisation of co-occurring foods. Co-occurrence food patterns have been used to assist in food portion size estimations from food images [10]. The consumption of a diversity of foods within a dietary pattern is often considered a marker of diet quality, providing a broader range of nutrients and biochemicals that impact human metabolism and gut microbiome composition [11]. Not only what we eat, but how or when we eat may also have health implications. Chrono-nutrition is a recently emerging field that considers the impact of circadian rhythm and its influence on dietary intake and natural metabolic oscillations [12]. Novel eating behaviour measures such as length of feeding window (time of first consumption of a food and drink, to the last) and breakfast consumers vs. breakfast skippers (identified here as a time period of > 6:00 am to < 11:00 am), may influence health-related outcomes such as body weight [13, 14].

Errors can arise across the dietary data pipeline from collection to processing [15]. Misreporting is a widely recognised bias in self-reported dietary data [16]. Less described are the flawed outcomes that can ensue from the method in which food records are coded. Errors can be induced from several areas, including poorly chosen food codes, number displacement, and difficulties in interpreting written participant records [15]. Inaccuracies in nutrient estimations related to static food composition tables can reflect variations in coder interpretation and entry of diet records, as well as the nutrient database in use, in opposition to tangible differences between individual nutrient intakes [15]. As such, enhanced efforts are required to minimise errors related to the collection and processing pipeline. STrengthening the Reporting of OBservational studies in Epidemiology – Nutritional Epidemiology (STROBE-nut) recommendations emphasise the importance of full-reporting of data-source measurement [17].

The TwinsUK cohort is one of the largest epidemiological research databases in the United Kingdom (UK) and the largest UK twin registry encompassing over 14,000 twins [18]. The twin structure of this cohort allows for the estimation of the genetic susceptibility of dietary aspects, with insight into the interplay between genes and diet crucial to understanding the pathophysiology of health and disease outcomes [19]. FFQs allowing for the characterisation of habitual diet and dietary patterns have traditionally been the sole method of dietary data collected in the TwinsUK cohort. In order to determine acute dietary intake and eating behaviours, EFR were collected on 2184 participants of this cohort between 2013 and 2017. This paper aims to; (1) describe data collection, processing and quality assessment of the EFR from the Twins UK cohort, (2) characterise nutrients, eating patterns and co-occurrence of foods, and (3) to test the heritability of diet behaviours, (4) and lastly to compare heritability of nutrient intake from the two dietary assessment methods (EFR and FFQ).

## Methods

### Study population and analysis

The participants were enrolled onto the TwinsUK registry, a registry of predominantly female, adult monozygotic and dizygotic twins in the UK. Ethical approval was obtained from St Thomas’ Hospital Research Ethics Committee with all subjects giving informed consent. This analysis included 1858 participants (1635 female and 223 male twins) between 18 and 89 years of age, who had previously completed one or more EFR between 2013 and 2017.

### Food record: exclusion criteria for descriptive and heritability analyses

**Fig. 1:**
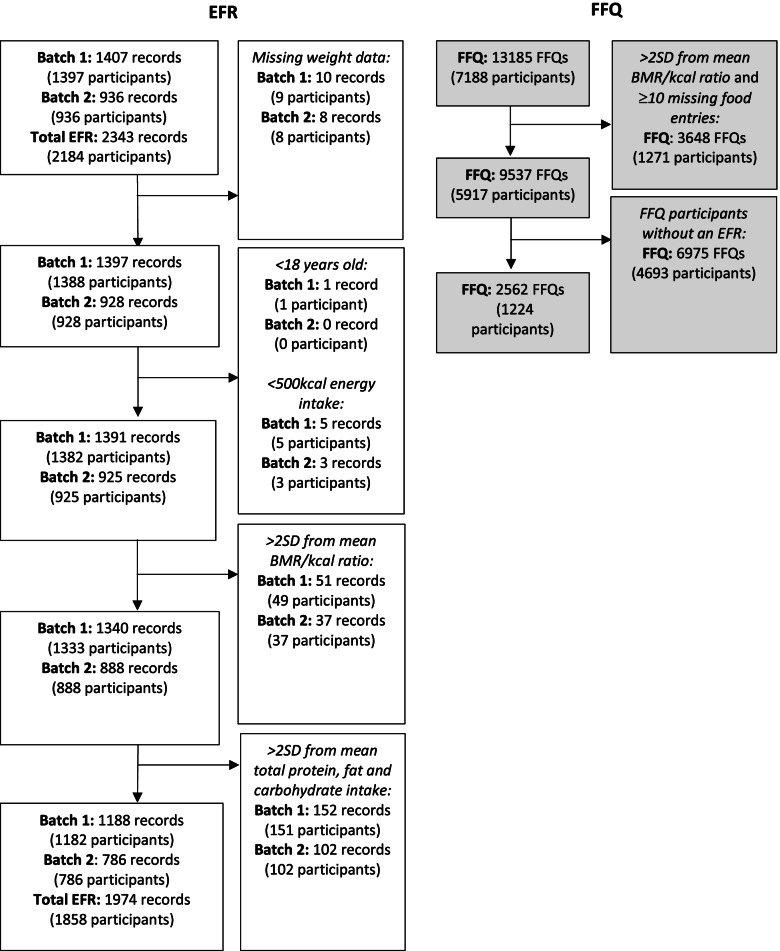
Flow chart of diet record (per batch) and FFQ inclusion. EFR; Estimated Food Record, FFQ; Food Frequency Questionnaire

EFR were excluded on a per batch basis according to the following criteria (Fig. 1); (1) < 500 kcal energy intake, considered physiologically unsustainable; (2) < 18 years old, (3) >2SD from mean of basal metabolic rate/calorie ratio calculated using the Harris-Benedict equation [20], in line with previous TwinsUK protocol for dietary assessments [21], (4) >2SD from the mean for protein, carbohydrate and/or fat to remove outliers after reviewing the distribution of the data. Those with missing demographic information (height, weight, age and sex) required for the Harris-Benedict equation were removed. One hundred fourteen participants (6.5%) had ≥2 EFR (2 Records = 112, 3 Records = 2), however these were not on consecutive days. One thousand two hundred twenty-four of the participants have completed FFQs, after exclusions (Fig. 1). EFRs and FFQs were not assessed at the same time periods.

### Collection and processing of estimated food records in the TwinsUK cohort

Two thousand three hundred forty-three estimated foods records were collected from 2184 participants in the TwinsUK cohort. Participants in the research cohort were requested to complete a food record if a stool sample was being collected. Structured open-ended paper-based EFR were provided with written instructions requesting information on portion size, mealtime, food item description, date and whether the participant considered it a typical day’s intake (Yes/No). Participants completed EFR any time prior to clinic visit, 28% of EFRs were completed on a weekend, and 72% on a weekday. Electronic formats of EFR were processed externally in two batches. Standard protocol was followed. The first batch (Batch 1) was composed of 1407 records (1397 participants, October 2013–January 2014) and were processed by University of East Anglia (WISP4, Tinuviel Software Ltd., McCance and Widdowson Food Composition Tables 5th and 6th Editions and all supplemental volumes [22]) with 60 dietary variables, defined as nutritive and non-nutritive food components. The second batch (Batch 2) was composed of 936 records (936 participants, January 2016–October 2017) and processed by Abacus Ltd. (Dietplan7, Foresoft Software Ltd., McCance and Widdowson database 7th Edition and the revised Composition of Foods Integrated Data Set) with 48 dietary variables. To assess quality of data processing, 30 randomly selected paper-scan EFR (> 10% of total number of EFR) were processed by a Research Dietitian using Dietplan7 nutrition software (Foresoft Software Ltd., McCance and Widdowson database 7th Edition and the revised Composition of Foods Integrated Data Set [23]). Where not described, portion sizes were allocated on the following basis; (1) if stipulated within the software, (2) according to *The Food Standards Agency Food Portion Sizes, 3rd Edition* [23], or (3) as detailed online by common supermarket suppliers. The STROBE-nut statement can be found in the supplementary materials.

### Collection and processing of food frequency questionnaire in the TwinsUK cohort

A 131-item Food Frequency Questionnaire (FFQ) was developed and validated against pre-established nutrient biomarkers for the European Prospective Investigation into Diet and Cancer (EPIC) Norfolk [24]. FFQs were administered to the Twins UK cohort in three batches; (1) from 1995 to 2001, (2) in 2007, and (3) from 2014 to 2015. FFQ procedures in the TwinsUK cohort have previously been described in detail elsewhere [21]. FFQs were excluded on a per batch basis if >2SD from mean of basal metabolic rate/calorie ratio calculated using the Harris-Benedict equation [20], if ≥10 incomplete food items in line with EPIC procedures [21, 24] and previous Twins UK practices [21], and if an EFR was not available (after the inclusion criteria) (Fig. 1). Nutrient intakes were transformed into an average daily intake, and residual energy adjusted per batch by mean energy intake.

### Assessing quality of food record participant entry

To assess participant recording completeness, 366 EFR were selected at random (> 15% of sample). Data with missing electronic scans of the paper entry (*n* = 7) were excluded. The remaining records (*n* = 359) were assessed via previous measures outlined by Goff et al. [25], applying a score of 0 = no information, 1 = incomplete information and 2 = complete information to variables portion size, food item description and time of consumption. A summed total score of ≥5 (out of 6) was deemed an acceptable quality on consensus of two dietitians.

### Food record: food item frequency and co-occurrence

For all analyses, R version 3.6.2 and RStudio version 1.1.463 were employed. From the individual food items recorded, 386 items were considered similar or exact matches to other food items (e.g. *‘Rice, Eggfried, Takeaway’, ‘Eggfried Rice, Takeaway*’). Therefore, a ‘food key’ was created for the McCance and Widdowson database (7th edition) and additional food items present within the EFR (total *n* = 4957). One thousand one hundred thirty-eight food items were manually aggregated into 542 ‘food key’ food items, overviewed by two dietitians. Nutrient composition of aggregated ‘food key’ items were determined by; (1) those with the largest number of nutrients complete were selected, (2) for those with the same number of complete nutrient values the mean was applied if within a 5% difference of variation. The food key condensed 2785 to 2399 food items across 1974 records filled by 1858 participants. Word clouds were generated for the 100 most frequently described individual food words and 100 most frequently consumed food item phrases (Supplementary Table 1). Two networks of commonly co-occurring foods were created using R package ‘igraph’ [26] after removing duplicate food items per participant, (1) all foods consumed within 1 day period (beverages milk, coffee, tea and wine were excluded), (2) those consumed at ‘breakfast’ (> 6:00 am and < 11:00 am) [27] (Supplementary Fig. 1).

### Food record vs. food frequency questionnaire

A Spearman’s two-sided correlation test was applied on the energy adjusted nutrient intake of participants who have both an EFR and a FFQ (*n* = 1224) for 44 nutrient variables (NAs excluded), after removing duplicate EFRs and FFQs, retaining the EFR and FFQ with the shortest time difference (mean 838 days, (±SD 1564) (Supplementary Table 2). Bland Altman plots were applied to energy intake, adjusted macronutrients and adjusted fibre (NSP) intake (AOAC fibre not available in FFQ output) to EFR and FFQs. After nutrient conversion to the same unit (grams), residual energy adjustment by the mean energy intake and normalisation of the data, Procrustes analysis (R package ‘Vegan’ [28]) was performed using Euclidean distances matrixes calculated on nutrient composition of EFR and FFQs; (1) 1224 participants with matching EFR and FFQs, (2) Between EFRs for those with > 1 EFR (*n* = 114), (3) Between FFQs for those with > 1 FFQ (*n* = 2356). Monte Carlo *p*-values for rotational agreement significance testing were determined from 999 permutations.

### Food record: impact of eating behaviours on nutrient intake and BMI

The impact of (1) breakfast consumers vs. skippers (breakfast consumed between 6:00 am and 11 am with a minimum intake of 100 kcal [27]), (2) length of eating window (time (hrs) from first consumption of food/drink to last) on energy, residual energy adjusted macronutrient and fibre intake [29], on BMI, were investigated using univariate analysis of variance (ANOVA) (null fit model, R package ‘lme4’ [30] and ‘stats’) adjusting for covariates of age (years), with sum of energy intake (kcal) as fixed effects and family and zygosity as random effects. EFR (*n* = 14) with < 4 h eating window were excluded due to likely implausible recording.

### Food record and FFQ: heritability

The twin aspect of the data available, with two dietary assessment methods, allowed for characterisation of the heritability of nutrient intakes by the EFR and by the FFQs (720 EFR, 720 participants, 203 monozygotic (MZ) pairs, 157 dizygotic (DZ) pairs) (Supplementary Table 3, Supplementary Fig. 2). The heritability of eating behaviours was explored through the EFR (1216 records, 1216 participants, 372 MZ pairs, 246 DZ pairs). Linear structural equation modelling was applied, adjusting for age, sex and BMI, to estimate the genetic and environmental components of variance in the nutrient intakes. Heritability assessment considers (A) additive genetic effects, (C) environmental effects in common, (E) environmental effects not in common/error; with univariate ACE, AE and CE models applied using the R package ‘mets’ [31]. CE models assume the complete correlation of additive genetic effects in MZ twins, with a correlation of 0.5 in DZ twins, and assumes that the common environmental effects contribute equally to MZ and DZ twin correlations. The lowest Akaike information criterion (AIC) was used to determine the best-fitting model.

## Results

### Quality of food record participant entry and data processing

EFR (*n* = 329) were assessed for quality of participant entry. 97% of EFR had complete information for meal timings, 3% contained incomplete meal timing information. 55% of portion sizes described by the participants contained complete information, with 45% of EFR containing incomplete information on portion sizes. 84% of the description of food items provided complete information, with 16% providing incomplete description of one or more food items. A quality score ≥ 5 (out of 6) was deemed acceptable quality, reviewed by two dietitians, and based on previous work by Goff et al. [25]. The mean quality score of the participants food record entry was 5.36 (±SD 0.76, range 3–6), with 75% of EFR considered an acceptable quality.

Participants are disproportionately twin females (88% vs. UK population 50.6% [32]), middle aged (58.65 years), Caucasian and slightly less overweight than the UK population average (BMI 25.9 kg/m^2^ vs. 27.5 kg/m^2^) [33] (Table 1). Sample macronutrient intakes through the EFRs (mean, ±SD) include energy (1847 kcal, ±SD 550.18), protein (79.93 g, ±SD 30.82), fibre (19.24 g, ±SD 9.08) carbohydrate (220 g, ±SD 220.38) and total fat (70.6 g, ±SD 28.04).

**Table 1:** Summary description of food record participants (*n* = 1858 participants)

*Characteristic*	*n = 1858*	*mean*	*±SD*	*median*	*range*	*min*	*max*
Age (yrs)^a^	–	58.65	14.07	61.46	71.36	18.59	89.96
Sex (M/F)	88% F, 12% M	–	–	–	–	–	–
BMI (kg/m^2^)^a^	–	25.94	4.98	25.04	39.26	15.93	55.18
Weight (kg)^a^	–	69.43	14.14	67.25	101.85	39.95	141.8
Ethnicity	97.1% Caucasian, 2.4% BME, 0.5% Missing			–	–	–	–
Zygosity (MZ/DZ)	242 DZ pairs, 361 MZ pairs	–	–	–	–	–	–
Protein (g)^a^	–	79.93	30.82	75.3	193.41	9.79	203.2
Fat (g)^a^	–	70.61	28.04	67.38	151.95	2.77	154.72
Carbohydrate (g)^a^	–	220.38	77.39	212.12	444.62	37.3	481.92
Fibre AOAC (g)^a^	–	19.24	9.08	18.09	77.2	0.99	78.19
Energy (kcal)^a^	–	1847.42	550.18	1806.15	3827.7	522.9	4350.6

^a^Summary description of participants, including at multiple timepoints for those with duplicate records (*n* = 1974 EFR). BMI, weight and age at time of food record completion

### Characterisation of acute dietary intake

**Fig. 2:**
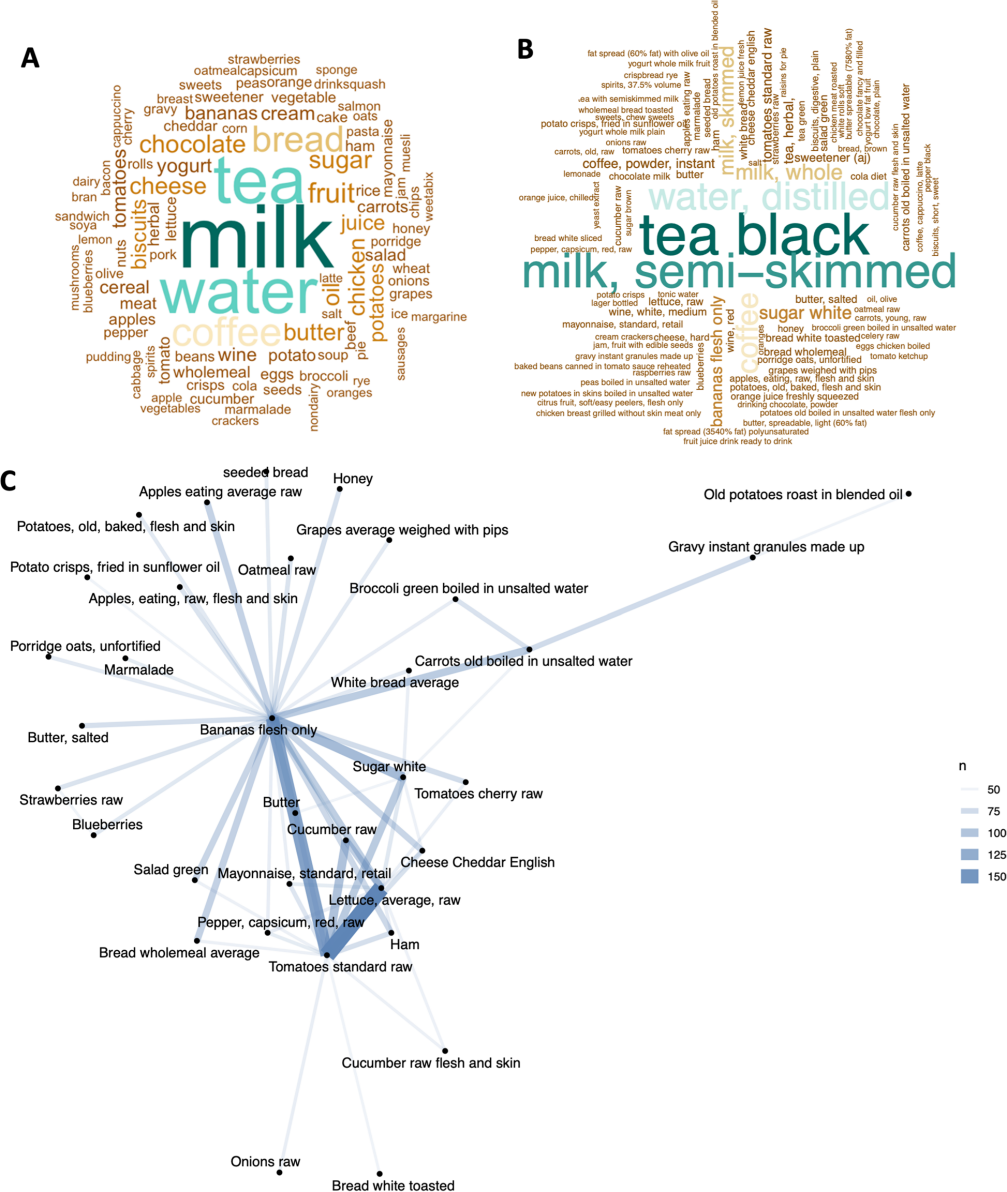
100 most frequently consumed food items, and food co-occurrence network of food pairings consumed within a food record; **A** 100 most frequently appearing word within food item descriptions, with the font size and colour depicting the importance of the food item **B** 100 most frequently appearing food item description, **C** co-occurrence network of most common food pairings within individual participants food records

A total of 2398 foods were consumed within 47,635 occasions in 1974 EFR. Tea and milk were the most frequently consumed food items, on both an individual and paired basis. ‘Tea, black, infusion, average’ comprised 8.27% (*n* = 3938) of food items, on average 1.99 occasions per food record, and ‘milk, semi-skimmed, pasteurised, average’ described 6.72% times (*n* = 3200), on average 1.62 occasions per food record. Two-word clouds were generated to display the frequency of food items; (1) the 100 most frequent single food and drink words (frequency range 118–6589) isolated from food item phrases (Fig. 2A, Supplemental Table 1), (2) the 100 most frequently consumed food item phrases, as described in the nutrition databases (frequency range 73–3938) (Fig. 2B, Supplemental Table 1). Food description pairings display the co-occurrence of food items consumed within a 24 h period (excluding beverages) (Fig. 2C), and within a typical breakfast period (Supplemental Fig. 1) (> 6:00, < 11:00 am). ‘Tea, black, infusion, average’ and ‘Milk, semi-skimmed, pasteurised, average’ were the top paired food and drink items (0.21%, *n* = 788). Excluding beverages, the top paired food items were ‘Tomatoes, standard, raw’ and ‘Lettuce, average, raw’ (0.05%, *n* = 167).

### Comparison of nutrient output via acute dietary intake vs. habitual measures

**Fig. 3:**
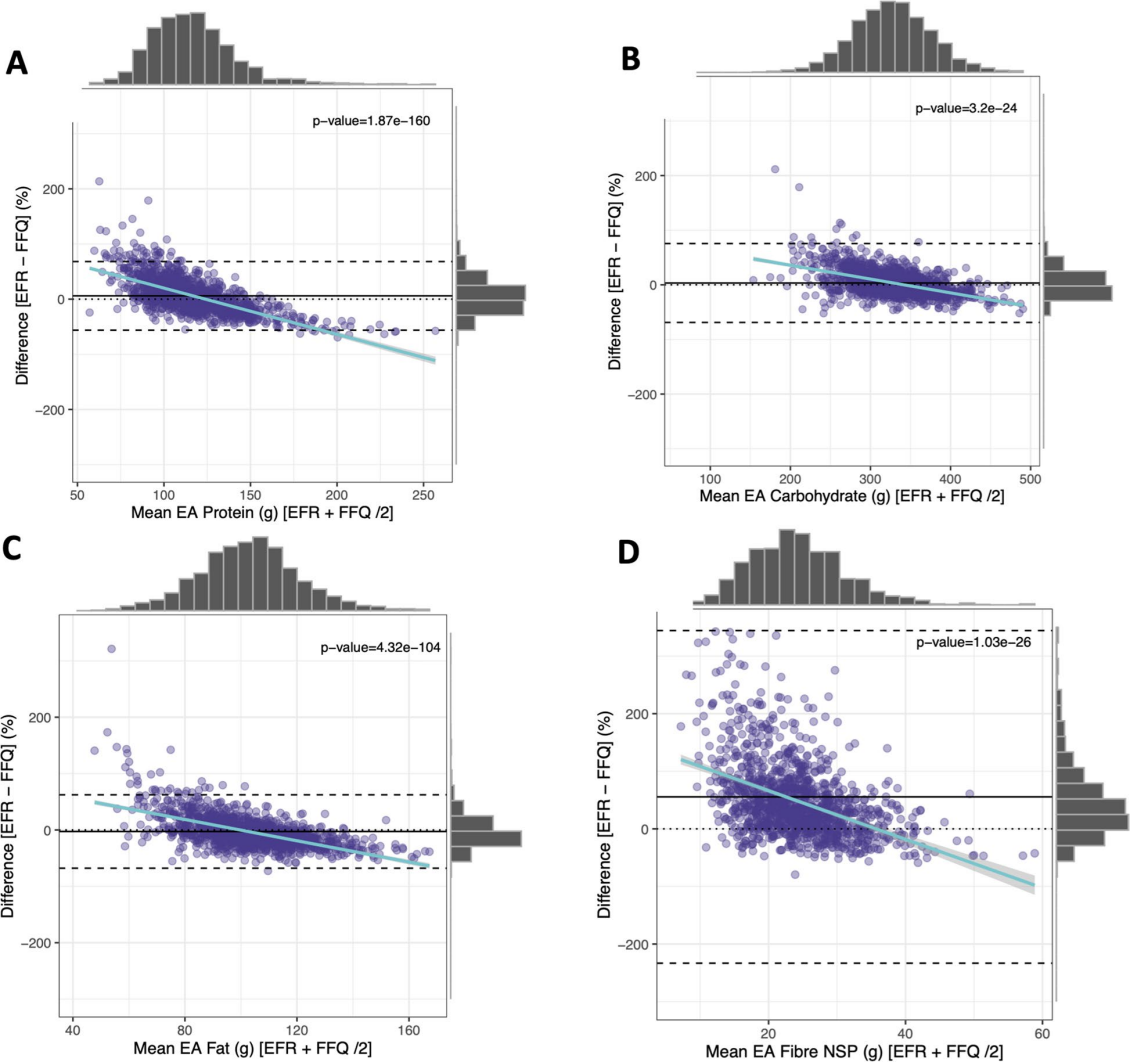
Bland Altman plot comparing residual energy adjusted macronutrient and fibre intake of EFR vs FFQs; **A** EA protein (g), **B** EA carbohydrate (g), **C** EA fat (g), **D** EA NSP fibre (g)

The agreement between two commonly employed dietary data assessment methods (EFR and FFQs) were assessed. Food frequency questionnaires are typically used to quantify habitual food intake via self-reported frequency of intake over a specified period, enabling calculation of an average day’s nutrient output. Bland Altman plots were used to show the mean agreement and 95% confidence intervals between EFR and FFQs (*n* = 1224) for macronutrients as a percentage of energy (Fig. 3). Energy intake of protein, carbohydrate and total fat intake (Fig. 3A, B and C) are predominantly tightly spread with minimal variation, with the majority of the results clustered around the mean difference line. However, there is greater variation at with lower intakes. NSP fibre intake measures displayed a greater variation of intakes via the EFR vs. the FFQ, with a wider spread of participants displaying a large percentage difference between the two dietary assessment methods (Fig. 3D).

Overall nutrient composition of participants diets collected at two different time points tended to be similar, despite the type of dietary assessment method employed. A spearman’s two-sided correlation test was performed on 44 residual energy adjusted variables between EFR and FFQs for 1224 participants, selecting those closest in date for those with multiple entries (Supplementary Table 2). Food record nutrient output and FFQ nutrient output are strongly correlated for 95% of dietary components (42 of 44), of which coefficients ranged from 0.09–0.46. The most strongly correlation dietary components were EA (energy adjusted) alcohol (g) and EA folate (ug).

**Fig. 4:**
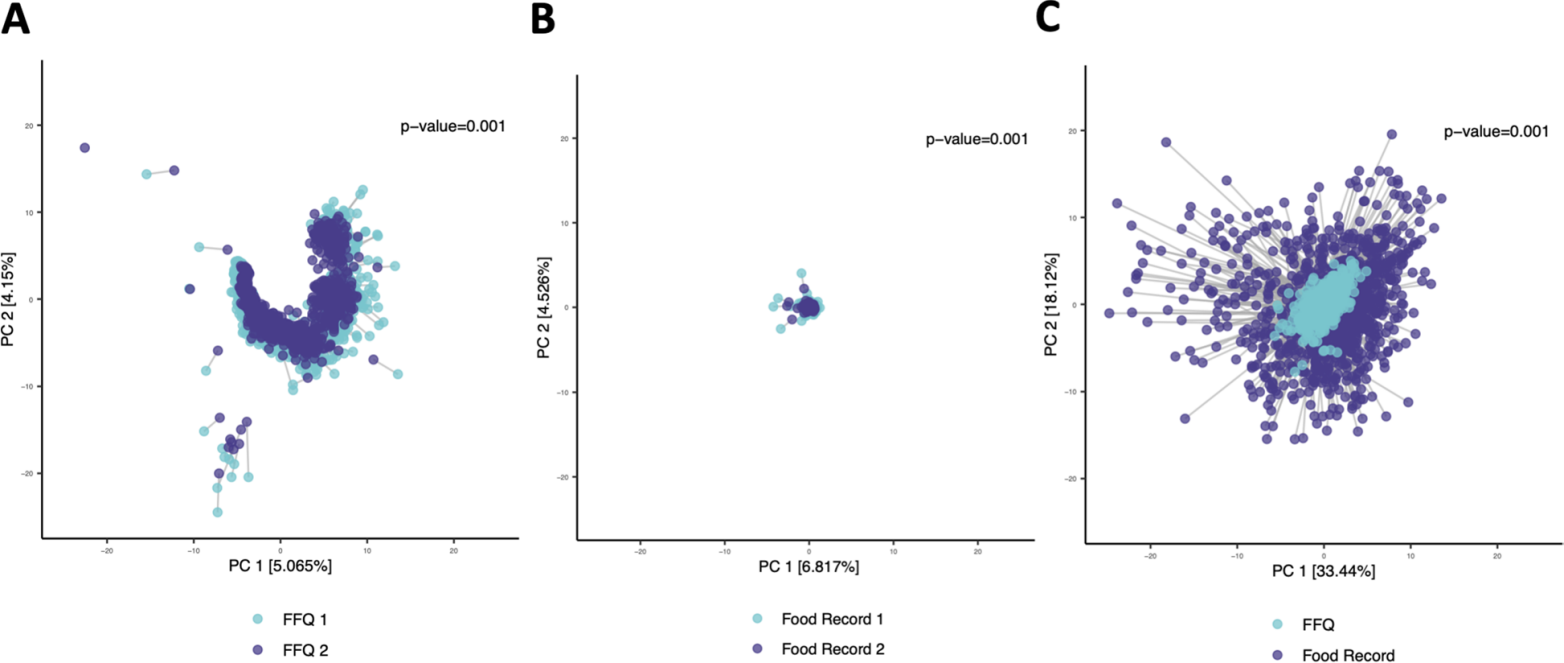
Procrustes analysis comparing the total intake (energy adjusted nutrient composition of 44 variables) between different dietary assessment methods for an individual, and between the same dietary assessment methods (taken at two different timepoints) for an individual. Multi-dimensional dietary variables are transformed into one datapoint. Each datapoint is the composition of a participant’s diet, the line connecting the diets of the same participant. The closer the distance between each point the more similar the dietary composition. **A** FFQ vs FFQ (*p* = 0.001) 2356 participants, **B** Food Record vs Food Record (*p* = 0.001) 114 participants, **C** Food record vs FFQ (*p* = 0.001) 1224 participants

A Procrustes analysis was performed to ascertain the distribution of participants’ nutrient intake as a shape or pattern, allowing intra- and inter-person comparison. The Procrustes analysis which applies multi-dimensional reduction to nutrient intake (Fig. 4A, B, C) reveals significant associations between and within the dietary assessment methods, with similar clustering patterns and minimal outliers between samples, particularly for between EFRs (Fig. 4B). These results suggest that participants consume similar nutrient intakes in acute (EFR) and long-term dietary settings (FFQs), with minimal variance.

### Characterisation of eating behaviour through estimated food records

Two eating behaviour measures were investigated using a linear mixed effect model after adjusting for covariates in the EFR; (1) breakfast consumption (or skipping) (breakfast: < 11 am, minimum intake 100 kcal), and (2) length of eating window defined as the period of time (in hours) between the first and last consumption of food or drink. In the EFR, breakfast consumers were found to consumed significantly higher quantities of energy (kcal) and EA macronutrients than breakfast skippers, respectively; energy (kcal) (mean 1874.67 (532.42) vs. 1700.45 (620.98), *p* = 5.99 × 10^− 7^), EA protein (mean 79.5 (±SD 24.45) vs. 74.84 (±SD 24.74), *p* = 0.01*), EA carbohydrate (g) (mean 201.66 (±SD 36.26) vs. 188.59 (±SD 34.63), *p* = 1.19 × 10^− 4^), and EA fibre (AOAC) (g) (mean 19.52 ± SD (8.02) vs. 16.73 (±SD 8.29), *p* = 6.26 × 10^− 6^), but lower intake of EA total fat (g) (mean 70.13 (±SD 16.89) vs. 16.73 (±SD 8.29), *p* = 0.005). However, despite this, consumption of breakfast was not found to be significantly associated with either BMI (*p* = 0.09) or weight (kg) (*p* = 0.09). However, breakfast skippers were found to have the longer eating window compared to breakfast consumers (mean respectively; 9.91 h (±SD 3.32), 8.26 h (±SD 2.42), *p* = 1.13 × 10^− 15^) (Table 2).

**Table 2:** Eating behaviour impact on nutrient intake and BMI status from EFR (*n* = 1845 participants, 1845 EFR)

	***Breakfast Consumers****(mean (*±*SD))*	***Breakfast Skippers****(mean (*±*SD))*	*Effect Size (+/−SE)*	*95% CI [LL, UL]*	*P Value*	***Eating Window****(mean (*±*SD))*	*Effect size (+/−SE)*	*95% CI [LL, UL]*	*P Value*
*n*	1623 participants, 1623 records	222 participants, 222 records	–	–	–	1845 participants, 1845 records	–	–	–
Sex (M/F)	M = 201, F = 1422	M = 22, F = 200	–	–	–	M = 223, F = 1622	–	–	–
Age (Years)	59.06 (13.72)	52.71 (15.83)	0.03 (0.12)	−0.20, 0.26	0.82	58.30 (14.14)	0.02 (0.26)	−0.49, 0.53	0.26
Weight (kg)	69.46 (13.99)	69.96 (15.52)	−1.12 (0.85)	−2.78, 0.54	0.19	69.52 (14.18)	0.21 (0.11)	−0.005, 0.42	0.05*
BMI (kg/m^2^)	25.91 (4.89)	26.16 (5.69)	−0.52 (0.31)	−1.12, 0.08	0.09	25.94 (4.99)	0.08 (0.04)	0.002, 0.16	0.04*
Eating Window (hrs)	8.26 (2.42)	9.91 (3.32)	−1.47 (0.18)	−1.82, − 1.12	1.13 × 10^− 15^*	8.46 (2.60)	–	–	–
Energy (kcal)	1874.67 (532.42)	1700.45 (620.98)	187.49 (37.49)	114.38, 260.60	5.99 × 10^−7^*	1853.71 (546.6)	2.48 (4.81)	−6.90, 11.86	0.61
EA Protein (g)	79.5 (24.45)	74.84 (24.74)	4.34 (1.72)	0.99, 7.69	0.01*	78.94 (24.53)	0.31 (0.22)	−0.12, 0.74	0.16
EA Carbohydrate (g)	201.66 (36.26)	188.59 (34.63)	12.72 (3.30)	6.29, 19.16	1.19 × 10^−4^*	200.19 (36.30)	−1.21 (0.44)	−2.07, −0.35	0.007*
EA Fat (g)	70.13 (16.89)	73.53 (18.87)	−3.69 (1.19)	−6.01, −1.37	0.005*	70.54 (17.17)	0.17 (0.16)	−0.14, 0.48	0.27
EA Fibre AOAC (g)	19.52 (8.02)	16.73 (8.29)	2.59 (0.57)	1.48, 3.70	6.26 × 10^−6^*	19.18 (8.10)	0.13 (0.07)	−0.01, 0.27	0.08

ANOVA (null fit model) adjusted for covariates age, sum of energy intake (kcal) (fixed effects), and family and zygosity (random effects)

*Statistically significant < 0.05

Investigating the impact of the length of eating window (hrs), a greater eating window was found to be significantly associated with both an increased weight (kg) (*p =* 0.05) and BMI status (*p* = 0.04), though the effect sizes were small (respectively; beta = 0.21 (±SD 0.11), beta = 0.08 (±SD 0.04)). The length of eating window was significantly associated with EA carbohydrate intake (g) (*p* = 0.007), though there was no significant association with energy intake, EA protein, EA fat or EA fibre intake.

### Heritability of eating behaviours and nutrient intake

**Fig. 5:**
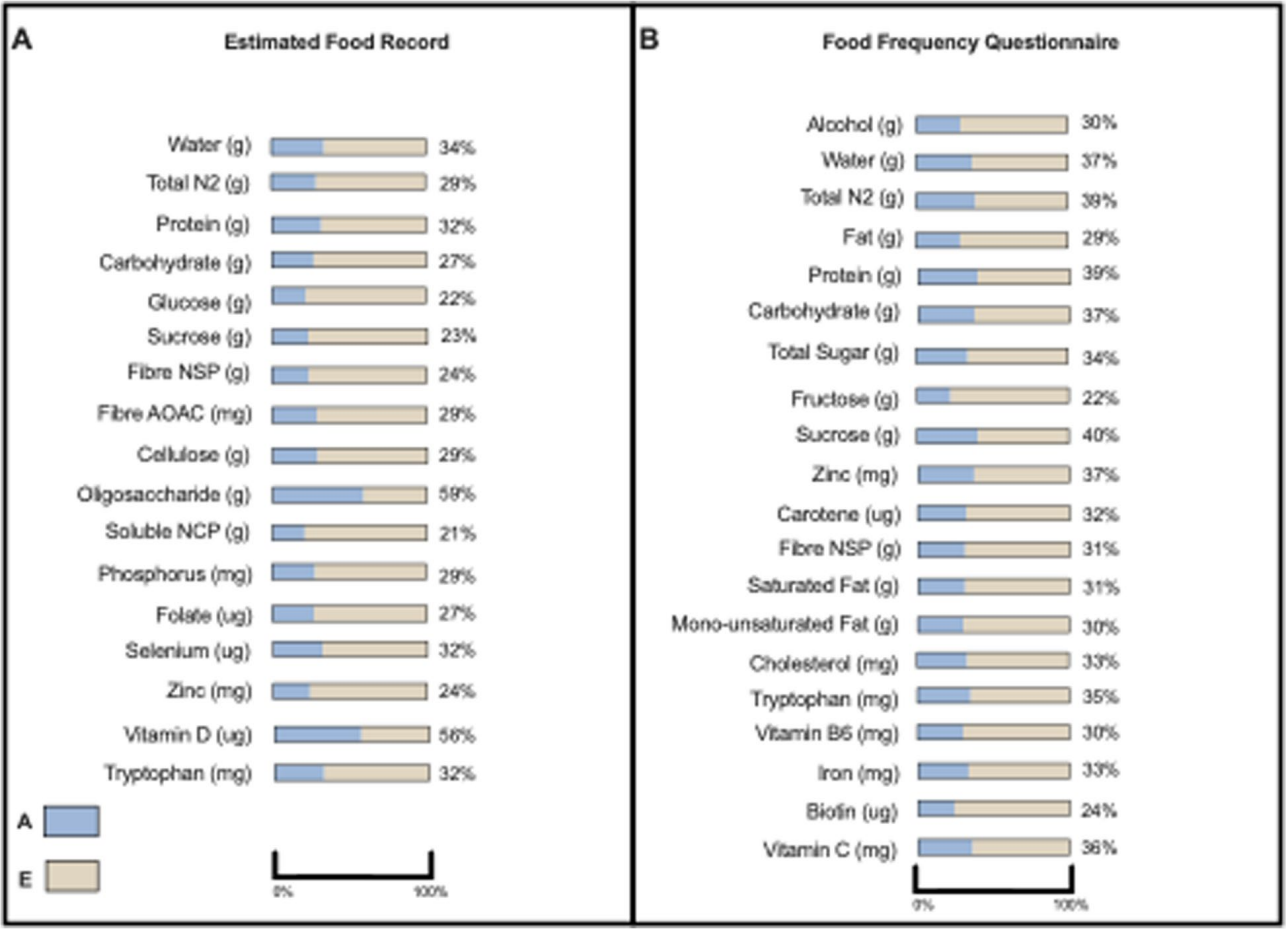
Moderate to great heritability of nutrients (*n* = 720) (> 20% AE model) estimated using linear structural equation modelling with considering **A** additive genetic effects, **C** environmental effects in common; **A** heritability of nutrients via EFR **B** heritability of nutrients via FFQ

The twin structure of the TwinUK cohort allows for the exploration of heritability of nutrients and eating behaviours using linear structural equation modelling (Table 3). The heritability of nutrients was estimated and compared between the estimated food record and FFQ nutrient outputs. From both dietary assessment methods, the nutrients were characterised to have a weak to moderate estimate for heritability (Fig. 5, Supplementary Table 3, Supplementary Fig. 2). The AE model was the best fitting model for majority of nutrients for both food record and FFQ output (respectively, 31 dietary variables, 27 dietary variables). Mean heritability (h^2^) estimates for food record were 13% (SD 15%), and 18% from the FFQ output (±SD 15%). In the food record, EA oligosaccharide (56%) and EA Vitamin D (56%) intakes were seen to be the most strongly heritable across the two dietary assessment methods.

**Table 3:** Heritability of eating behaviours from food record (*n* = 1216 participants, 1216 EFR)

*Eating Behaviour*	*Model of Best Fit*	*A[95%CI]*	*E[95%CI]*
Breakfast consumers	AE	0.11[0.02;0.21]	0.89[0.79;0.98]
Eating window (hrs)	AE	0.33[0.24;0.41]	0.59[0.76;0.76]

From the FFQ, EA Vitamin D was not estimated to be heritable with the predominant effect on variance thought to be environmental. EA oligosaccharide intake is not a measured output for the FFQ; the two most strongly heritable components from the FFQ with moderate heritability were EA protein (39%) and EA N_2_ (39%). The best fitting model for eating window length and consumption of breakfast was the AE model, suggesting an aspect of heritability. Eating window length was found to be moderately heritable (33%), while breakfast consumption only weakly so (11%).

## Discussion

This is the first presentation of the EFR from the TwinsUK cohort, facilitating the characterisation of data procedures, quality assessment and characterisation of acute dietary intake and eating behaviours. Transparency in data methodologies and documentation undoubtably improves scientific rigor and credibility, advancing nutrition research [34]. Dietary data is generated via a stepwise process including collection and coding [24]; sources of discrepancies can therefore occur at any point in the pipeline from participant recording of dietary intake to the outputs of the nutrition analysis software [35]. After a quality assessment, the EFR were considered of predominantly an acceptable quality, according to parameters conferred by Goff et al. The macronutrient output predominantly reflected that of the wider population (UK National Diet and Nutrition Survey (NDNS), Years 7–8, 19–64 years old [36], with agreement between EFRs and FFQs though increased differences at lower intakes possibly related to differences in methods (gathering acute vs. habitual intakes). Portion size descriptions within the EFRs were limited with 45% containing incomplete information. Omission of foods and errors in portion size estimations have been associated with errors in coding during dietary data processing [7]. Kolar et al. evaluated 3-day self-administered diet diaries, where participants were reported to have missing portion size information for 3% of the food items described, with 8% of the portion sizes of foods incompletely described [37]. However, study participants were given instructions for recording food intake together with a 12-page serving size booklet containing photographs and measurement tools to aid and prompt complete and accurate data recording. Interestingly, the researchers noted that there was a trend relating an increased number of missing portion sizes with a greater BMI [37]. This may be related to internal biases such as social desirability bias and recall bias [3].

Due to their less labour intensive nature, self-reported dietary intake via prospective or recall methods are frequently employed in nutritional epidemiological investigations [38]. Overall, EFR are considered a detailed assessment method, providing good estimation of the energy intake and the intake of most nutrients, foods and food groups [3], while allowing characterisation of eating behaviours, cultural food choices and food combinations. EFRs are less burdensome to participants than 24-h recalls as they allow for estimation of portion sizes rather than weighing of food items [39]. In this study, we observed that most nutrient outputs of EFR and FFQs were significantly associated, on both an individual level and as a nutrient pattern (Procrustes analysis). Previous work by Mazzeo et al. has described a moderate agreement between the EPIC FFQ and a 7-day weighed food diary, with 70% of nutrients displaying a Spearman’s correlation co-efficient of 0.30 and above [40]. The collection of multiple consecutive food records is well acknowledged as being the preferable measure of acute dietary intake to account for variation in diet [3]. In this investigation, the use of a one-day food record was found to reflect similar nutrient intakes to the output described through an FFQ, though displaying a lower agreement (0.40) between the two methods than the previously described study. A comparison of different methods of collecting food records in the EPIC cohort displayed that self-reported 7-day open-ended food records had the closest agreement to 16 days of weighed food intake, with the highest correlation with biomarkers when compared to a 24 h recall and food frequency questionnaire [41]. However, participant and researcher burden must be considered; increased days may lead to diminished accuracy of dietary data relative to study fatigue.

EFR are a rich source of data that enable the characterisation of eating behaviours. Nutrition intervention strategies frequently solely focus on dietary components, however ‘how’ we eat may also influence health. Observational studies have characterised breakfast consumption as associated with a lower BMI and may be protective against weight gain [42, 43]. In a 2019 systematic review and meta-analysis, researchers concluded that while breakfast consumption was associated with increase in total daily calories, there was only a nominal difference in weight between those who consumed breakfast vs. skipped breakfast (respective mean difference 0.44 kg) [13]. In this investigation, breakfast consumption was not significantly associated with a lesser or greater BMI status or weight, despite a significantly higher energy (kcal) intake for breakfast consumers. Interestingly, a longer eating window was significantly associated with a higher BMI and weight. Breakfast skippers were found to have longer eating window than breakfast consumers, which could explain, in part, the lack of significant discrepancy between the consumers’ and skippers’ weight and BMI status. Time-restricted feeding, such as consuming daily food intake within an 8–10 h eating window, has been reported to improve glucose tolerance, though more research is warranted in humans [44–46].

Heritability estimates assess the proportion of phenotypic variation related to genetic factors. In humans, the estimation of heritability can assist in establishing the genetic aspects of biological and behavioural traits and disease states, such as height [47] and schizophrenia [48]. Understanding the influence of genetics on diet and eating behaviours may aid understanding of their ability to be modulated for therapeutic health strategies, with unhealthful dietary habits considered leading risk factors for many disease states. Previous work within the TwinsUK cohort established a genetic link to dietary patterns that accounted for 22% of total variance with estimates ranging from 41 to 48%. Individual foods such as fruit and vegetable intakes (49%), garlic (46%), coffee (41%) were also found to have strongly heritable components [21], possibly related to olfactory receptor loci [49], however this was not explored in this investigation. Heritability of nutrient intake may be influenced by dietary assessment method, as characterised in this study, with FFQs typically considered a more appropriate method for investigating nutrient heritability as a measure of habitual diet. As expected, the results reflected that heritability associations with a FFQ were typically broader and stronger than through an EFR, despite a higher number of nutrients measured in the EFR. Preliminary evidence suggests that there may be genetic aspects to eating behaviours, though the number of published studies are limited. The genetic influence of food timing has been previously characterised in 53 adult Spanish female twin pairs, with the timing of breakfast found to be strongly heritable (56%), with the midpoint of food intake found to be even more so (64%). In the UK Biobank, 6 genetic variants have been identified for breakfast skipping which regulate the pace of the circadian clock, suggesting a possible beneficial role of breakfast consumption [50]. This may be supported by our findings of the moderate heritability of the length of eating window (33%), and mild heritability of breakfast consumption (11%), suggesting a low genetic aspect to these phenotypes. Therapeutic dietary interventions may be more effective through targeting traits which have a predominantly environmental influence. While this data provides rich insight into the food and nutrient intake, and eating behaviours of this sample population, the cohort is predominantly female, of older age and on average an overweight BMI status which may limit the extrapolation of findings to the wider population. Therefore, replication is required in an independent cohort.

This TwinsUK EFR dataset allows for the exploration of the impact of acute diet, and new insights into eating behaviours and co-occurrences of food items on health and disease states in this twin population. However, the predominantly female, older-aged, Caucasian nature of the research cohort should be considered, as may not represent the diversity of the UK population. While the majority of EFR were an acceptable quality, further data improvements may have been possible with the implementation of standard operations procedures for each stage in the data collection and processing pipeline, and are recommended for future dietary data investigations in this cohort. Likewise, the poor portion size estimation should be addressed, whether through provision of a portion size guide or a technology assisted approach such as a mobile application. Portion size missingness by BMI status was not explored in this study. The FFQ aims to estimate usual intake, while EFRs captures acute dietary intake which is typically more highly variable in nature and not an estimate of usual intake, therefore some level of measurement error may have been induced [51]. Only 1 day of EFR was collected at a time, to reduce participant burden. Typically, multiple days of EFRs are recommended in order to account for daily variation [39], however only one EFR was collected in this study. A comparison of methods found that 7-day EFRs were in closest agreement with 16 days of weighed food intake in the UK arm of the EPIC study, and next closest in similarity with biomarkers, compared to a FFQ and 24-h recall [41]. EFRs also allow for the assessment of eating frequency and snacking and meal patterns, while not explored here, may warrant further investigation in this cohort. Application of statistical modelling to minimise measurement error could be applied to future EFRs particularly where multiple EFRs are available for a higher proportion of the sample.

## Conclusion

EFR are affordable, easy to use, non-invasive and flexible by means of implementation (e.g. electronic, paper, online); suitable for large studies. Due to the open-ended nature of an EFR, EFR are better able to capture the complexities and variability in individual diets in comparison to more structured methods including insight into, for example, eating behaviours and the co-occurrences of food items. This study characterises the quality of the EFR from the TwinsUK cohort as acceptable. However, increased support for participants on reporting portion sizes of food and drink intake may be required, such as through the use of food quantity guides, to facilitate improved consistency in data quality. The impact of breakfast consumption vs. breakfast skipping on health is widely debated in the public arena however this study suggests that other behaviours, such as length of eating window, may be equally aligned to outcomes such as weight and BMI status, requiring further exploration. Likewise, the moderate heritability of the length of eating window and breakfast consumption are indicative of the need for discrimination and quantification of genetic and environmental influences of eating behaviours, and not just dietary intake. This study offers a preliminary insight into the rich potential of this dataset. The TwinsUK EFR allow for exploration into acute dietary intake, eating behaviours, heritability and co-occurring food choices, and may provide a useful adjunct to habitual diet measures in diet-disease investigations.

## Supplementary Information

**Additional file 1. Supplementary Table 1. Frequency of food words and food phrases.**

**Additional file 2: Supplementary Figure 1.** Co-occurrence network of breakfast.

**Additional file 3: Supplementary Table 2.** Two-sided Spearman’s correlation test comparing energy adjusted nutrient output of EFR vs FFQ (*n* = 1224).

**Additional file 4: Supplementary Table 3.** Heritability of nutrients, FFQ vs food record.

**Additional file 5: Supplementary Figure 2.** Heritability of nutrients, FFQ vs. EFR.

**Additional file 6: Table 1.** STROBE-nut: An extension of the STROBE statement for nutritional epidemiology.

## Data Availability

Data is available to researchers on request through TREC at the Department of Twins Research and Epidemiology, King’s College London. Further information can also be found on the TwinsUK website (www.twinsuk.ac.uk/data-access).

## Abbreviations

EFR: Estimated Food Records
FFQ: Food Frequency Questionnaire
QA: Quality Assessment
NDNS: UK National Diet and Nutrition Survey
BMI: Body Mass Index
EA: Energy Adjusted
BME: Black and Mixed Ethnic Minorities
