# Supplementary figures and images for: Characterisation, procedures and heritability of acute dietary intake in the Twins UK cohort: an observational study
Source: Nutr J. 2022 Feb 27;21:13. doi: 10.1186/s12937-022-00763-3 (PMC8883626; doi:10.1186/s12937-022-00763-3)

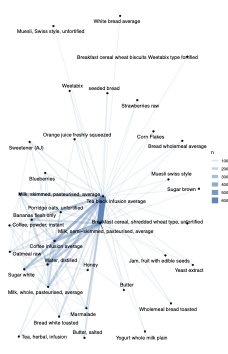

Supplement: Supplementary file 2 — Additional file 2: Supplementary Figure 1. Co-occurrence network of breakfast. [file 12937_2022_763_MOESM2_ESM.png]

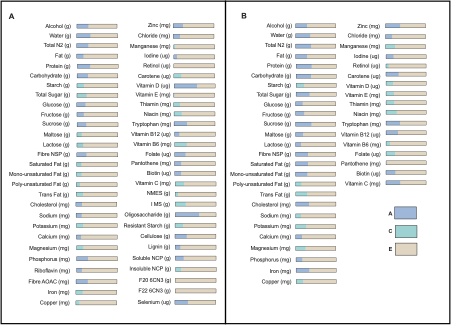

Supplement: Supplementary file 5 — Additional file 5: Supplementary Figure 2. Heritability of nutrients, FFQ vs. EFR. [file 12937_2022_763_MOESM5_ESM.png]
