# Supplementary material for: Characterisation, procedures and heritability of acute dietary intake in the Twins UK cohort: an observational study
Source: Nutr J. 2022 Feb 27;21:13. doi: 10.1186/s12937-022-00763-3 (PMC8883626; doi:10.1186/s12937-022-00763-3)
Supplement: Supplementary file 3 — Additional file 3: Supplementary Table 2. Two-sided Spearman’s correlation test comparing energy adjusted nutrient output of EFR vs FFQ (n = 1224). [file 12937_2022_763_MOESM3_ESM.docx]

***Supplementary Table 2****. Two-sided Spearman’s correlation test comparing energy adjusted nutrient output of EFR vs FFQ (n=*1224*)*

| *Variable* | *Estimate* | *P value* |
| --- | --- | --- |
| Alcohol g | 0.46 | 1.16 X10^-47^ |
| Water g | 0.25 | 4.63 X10^-19^ |
| Tot N2 g | 0.20 | 5.28 X10^-12^ |
| Protein g | 0.21 | 2.30 X10^-13^ |
| Fat g | 0.22 | 2.08 X10^-14^ |
| CHO mg | 0.26 | 1.19 X10^-20^ |
| Starch mg | 0.20 | 2.03 X10^-12^ |
| Total Sugar mg | 0.31 | 1.97 X10^-28^ |
| Glucose g | 0.29 | 7.81 X10^-26^ |
| Fructose g | 0.30 | 1.60 X10^-26^ |
| Sucrose mg | 0.29 | 7.92 X10^-25^ |
| Maltose mg | 0.17 | 6.37 X10^-09^ |
| Lactose mg | 0.37 | 1.21 X10^-40^ |
| NSP Fibre g | 0.31 | 2.00 X10^-28^ |
| Sat fats g | 0.22 | 1.34 X10^-14^ |
| Mono-unsaturated fats g | 0.18 | 3.57 X10^-10^ |
| Poly-unsaturated fats g | 0.14 | 9.92 X10^-7^ |
| Trans fats g | 0.13 | 3.22 X10^-6^ |
| Cholesterol mg | 0.23 | 1.43 X10^-16^ |
| Sodium mg | 0.04 | 0.171 |
| Potassium mg | 0.21 | 3.48 X10^-13^ |
| Calcium mg | 0.24 | 7.13 X10^-18^ |
| Magnesium mg | 0.26 | 2.42 X10^-20^ |
| Phosphorus mg | 0.25 | 1.30 X10^-18^ |
| Iron mg | 0.20 | 4.85 X10^-13^ |
| Copper mg | 0.11 | 1.124 X10^-4^ |
| Zinc mg | 0.14 | 1.18 X10^-6^ |
| Chloride mg | 0.04 | 0.139 |
| Manganese mg | 0.27 | 1.23 X10^-21^ |
| Iodine ug | 0.27 | 6.76 X10^-22^ |
| Retinol ug | 0.11 | 1.12 X10^-4^ |
| Carotene ug | 0.24 | 5.81 X10^-17^ |
| Vitamin D ug | 0.16 | 2.96 X10^-8^ |
| Vitamin E mg | 0.09 | 0.001 |
| Thiamine mg | 0.21 | 8.92 X10^-14^ |
| Riboflavin mg | 0.34 | 1.32 X10^-34^ |
| Niacin mg | 0.20 | 5.99 X10^-13^ |
| Tryptophan 60 mg | 0.19 | 3.37 X10^-11^ |
| Vit B6 mg | 0.22 | 2.40 X10^-14^ |
| Vit B12 ug | 0.17 | 2.14 X10^-9^ |
| Folate ug | 0.31 | 4.30 X10^-28^ |
| Pantothen mg | 0.25 | 7.97 X10^-19^ |
| Biotin ug | 0.28 | 3.15 X10^-24^ |
| Vitamin C mg | 0.23 | 3.53 X10^-16^ |
