# Supplementary material for: Characterisation, procedures and heritability of acute dietary intake in the Twins UK cohort: an observational study
Source: Nutr J. 2022 Feb 27;21:13. doi: 10.1186/s12937-022-00763-3 (PMC8883626; doi:10.1186/s12937-022-00763-3)
Supplement: Supplementary file 4 — Additional file 4: Supplementary Table 3. Heritability of nutrients, FFQ vs food record. [file 12937_2022_763_MOESM4_ESM.docx]

***Supplementary Table 3.*** *Heritability of nutrients, FFQ vs food record*

|  | *Food Record* | | | | | | *FFQ* | | | | | | | |
| --- | --- | --- | --- | --- | --- | --- | --- | --- | --- | --- | --- | --- | --- | --- |
| *Biochemical* | *Model of Best Fit* | *A[95%CI]* | *C[95%CI]* | | *E[95%CI]* | | *Model of Best Fit* | | *A[95%CI]* | | *C[95%CI]* | | *E[95%CI]* | |
| EA Alcohol (g) | CE | 0 | 0.21[0.09;0.32] | | 0.79[0.68;0.91] | | AE | | 0.30[0.16;0.38] | | 0 | | 0.73[0.62;0.84] | |
| EA Water (g) | AE | 0.34[0.22;0.46] | 0 | | 0.66[0.54;0.78] | | AE | | 0.37[0.27;0.47] | | 0 | | 0.63[0.53;0.73] | |
| EA Total N2 (g) | AE | 0.29[0.19;0.40] | 0 | | 0.71[0.60;0.81] | | AE | | 0.39[0.28;0.50] | | 0 | | 0.61[0.50;0.72] | |
| EA Protein (g) | AE | 0.32[0.21;0.42] | 0 | | 0.68[0.58;0.79] | | AE | | 0.39[0.28;0.50] | | 0 | | 0.61[0.50;0.72] | |
| EA Fat (g) | AE | 0.17[0.05;0.29] | 0 | | 0.83[0.71;0.95] | | AE | | 0.29[0.17;0.41] | | 0 | | 0.71[0.59;0.83] | |
| EA Carbohydrate (g) | AE | 0.27[0.16;0.38] | 0 | | 0.73[0.62;0.84] | | AE | | 0.37[0.26;0.49] | | 0 | | 0.63[0.51;0.74] | |
| EA Starch (g) | CE | 0 | 0.17[0.07;0.27] | | 0.83[0.73;0.93] | | CE | | 0 | | 0.20[0.10;0.30] | | 0.80[0.70;0.90] | |
| EA Total Sugar (g) | CE | 0 | 0.24[0.14;0.33] | | 0.76[0.67;0.86] | | AE | | 0.34[0.22;0.46] | | 0 | | 0.66[0.54;0.78] | |
| EA Glucose (g) | AE | 0.22[0.10;0.34] | 0 | | 0.78[0.66;0.90] | | AE | | 0.18[0.06;0.31] | | 0 | | 0.82[0.69;0.94] | |
| EA Fructose (g) | AE | 0.17[0.06;0.27] | 0 | | 0.83[0.73;0.94] | | AE | | 0.22[0.09;0.34] | | 0 | | 0.78[0.66;0.91] | |
| EA Sucrose (g) | AE | 0.23[0.11;0.35] | 0 | | 0.77[0.65;0.89] | | AE | | 0.40[0.30;0.51] | | 0 | | 0.60[0.49;0.70] | |
| EA Maltose (g) | CE | 0 | 0.12[0.02;0.23] | | 0.88[0.78;0.98] | | AE | | 0.19[0.07;0.31] | | 0 | | 0.81[0.69;0.93] | |
| EA Lactose (g) | CE | 0 | 0.16[0.06;0.26] | | 0.84[0.74;0.94] | | AE | | 0.14[0.03;0.26] | | 0 | | 0.86[0.74;0.97] | |
| EA Fibre NSP (g) | AE | 0.24[0.12;0.36] | 0 | | 0.76[0.64;0.88] | | AE | | 0.31[0.20;0.43] | | 0 | | 0.69[0.69;0.80] | |
| EA Saturated Fats (g) | CE | 0 | 0.12[0.02;0.22] | | 0.88[0.78;0.98] | | AE | | 0.31[0.20;0.42] | | 0 | | 0.69[0.58;0.80] | |
| EA Mono Unsaturated Fats (g) | CE | 0 | 0.13[0.03;0.23] | | 0.87[0.77;0.97] | | AE | | 0.30[0.18;0.41] | | 0 | | 0.70[0.59;0.82] | |
| EA Poly Unsaturated Fats (g) | CE | 0 | 0.09[-0.02;0.19] | | 0.91[0.81;1.02] | | CE | | 0 | | 0.15[0.05;0.25] | | 0.85[0.75;0.95] | |
| EA Trans Fats (g) | CE | 0 | 0.17[0.07;0.27] | | 0.83[0.73;0.93] | | CE | | 0 | | 0.29[0.19;0.38] | | 0.71[0.62;0.81] | |
| EA Cholesterol (mg) | AE | 0.15[0.03;0.26] | 0 | | 0.85[0.74;0.97] | | AE | | 0.33[0.22;0.44] | | 0 | | 0.67[0.56;0.78] | |
| EA Sodium (mg) | AE | 0.14[0.01;0.27] | 0 | | 0.86[0.73;0.99] | | CE | | 0 | | 0.14[0.04;0.24] | | 0.86[0.76;0.96] | |
| EA Potassium (mg) | CE | 0 | 0.17[0.07;0.27] | | 0.83[0.73;0.93] | | CE | | 0 | | 0.27[0.18;0.37] | | 0.73[0.63;0.82] | |
| EA Calcium (mg) | AE | 0.12[-0.02;0.23] | 0 | | 0.89[0.77;1.02] | | AE | | 0.16[0.04;0.28] | | 0 | | 0.84[0.72;0.96] | |
| EA Magnesium (mg) | CE | 0 | 0.17[0.07;0.27] | | 0.83[0.73;0.93] | | CE | | 0 | | 0.25[0.15;0.35] | | 0.75[0.65;0.85] | |
| EA Phosphorus (mg) | AE | 0.29[0.14;0.35] | 0 | | 0.75[0.65;0.86] | | AE | | 0.16[0.04;0.28] | | 0 | | 0.84[0.72;0.96] | |
| EA Iron (mg) | CE | 0 | 0.17[0.07;0.27] | | 0.83[0.73;0.93] | | AE | | 0.33[0.22;0.45] | | 0 | | 0.66[0.55;0.78] | |
| EA Copper (mg) | CE | 0 | 0.09[-0.01;0.19] | | 0.91[0.81;1.01] | | CE | | 0 | | 0.17[0.27;0.83] | | 0.83[0.73;0.93] | |
| EA Zinc (mg) | AE | 0.24[0.14;0.35] | 0 | | 0.76[0.65;0.86] | | AE | | 0.37[0.26;0.48] | | 0 | | 0.63[0.74;0.37] | |
| EA Chloride (mg) | AE | 0.17[0.03;0.30] | 0 | | 0.83[0.70;0.97] | | AE | | 0.17[0.05;0.29] | | 0 | | 0.83[0.71;0.95] | |
| EA Manganese (mg) | CE | 0 | 0.04[-0.07;0.14] | | 0.96[0.86;1.07] | | CE | | 0 | | 0.24[0.14;0.76] | | 0.76[0.66;0.86] | |
| EA Iodine (ug) | AE | 0.08[-0.05;0.21] | 0 | | 0.92[0.79;1.05] | | AE | | 0.19[0.08;0.31] | | 0 | | 0.81[0.69;0.92] | |
| EA Retinol (ug) | AE | 1.83 x10^-5^[-0.02;0.02] | 0 | | 0.10[0.98;1.02] | | CE | | 0 | | 0.07[-0.04;0.17] | | 0.93[0.83;1.04] | |
| EA Carotene (ug) | CE | 0 | 0.189[0.09;0.29] | | 0.81[0.71;0.91] | | AE | | 0.32[0.21;0.44] | | 0 | | 0.68[0.56;0.79] | |
| EA Vitamin D (ug) | AE | 0.56[0.46;0.66] | 0 | | 0.44[0.34;0.54] | | CE | | 0 | | 0.18[0.08;0.28] | | 0.82[0.72;0.92] | |
| EA Vitamin E (mg) | AE | 0.02[-0.07;0.12] | 0 | | 0.98[0.88;1.07] | | CE | | 0 | | 0.20[0.10;0.30] | | 0.80[0.70;0.90] | |
| EA Thiamin (mg) | CE | 0 | 0.16[0.05;0.26] | | 0.84[0.74;0.95] | | CE | | 0 | | 0.21[0.11;0.31] | | 0.79[0.69;0.89] | |
| EA Niacin (mg) | CE | 0 | 0.19[0.10;0.29] | | 0.81[0.71;0.90] | | CE | | 0 | | 0.27[0.18;0.37] | | 0.73[0.63;0.82] | |
| EA Trypt.60. (mg) | AE | 0.32[0.22;0.42] | 0 | | 0.68[0.58;0.78] | | AE | | 0.35[0.23;0.47] | | 0 | | 0.65[0.53;0.77] | |
| EA Vitamin B6 (mg) | CE | 0 | 0.30[0.20;0.40] | | 0.70[0.61;0.80] | | AE | | 0.30[0.19;0.42] | | 0 | | 0.70[0.58;0.81] | |
| EA Vitamin B12 (ug) | AE | 0.12[-0.01;0.26] | 0 | | 0.88[0.74;1.01] | | CE | | 0 | | 0.10[6.25 x10^-4^;0.21] | | 0.90[0.79;1.00] | |
| EA Folate (ug) | AE | 0.27[0.16;0.39] | 0 | | 0.73[0.61;0.84] | | CE | | 0 | | 0.23[0.13;0.33] | | 0.77[0.67;0.87] | |
| EA Pantothene (mg) | AE | 0.17[0.05;0.30] | 0 | | 0.83[0.70;0.95] | | AE | | 0.01[-0.09;0.11] | | 0 | | 0.99[0.89;1.09] | |
| EA Biotin (ug) | AE | 0.16[0.06;0.27] | 0 | | 0.84[0.73;0.94] | | AE | | 0.24[0.12;0.35] | | 0 | | 0.76[0.65;0.88] | |
| EA Vitamin C (mg) | CE | 0 | 0.22[0.12;0.31] | | 0.78[0.69;0.88] | | AE | | 0.36[0.24;0.48] | | 0 | | 0.64[0.52;0.76] | |
| EA NMES (g) | CE | 0 | 0.07[-0.04;0.19] | | 0.93[0.81;1.04] | | - | | - | | - | | - | |
| EA I.MS (g) | CE | 0 | 0.26[0.16;0.37] | | 0.74[0.63;0.84] | | - | | - | | - | | - | |
| EA Oligosacc (g) | AE | 0.59[0.43;0.75] | 0 | | 0.41[0.25;0.57] | | - | | - | | - | | - | |
| EA Resistant Starch (g) | CE | 0 | 0.20[0.09;0.31] | | 0.80[0.69;0.91] | | - | | - | | - | | - | |
| EA Cellulose (g) | AE | 0.29 [0.61;0.41] | 0 | | 0.71[0.59;0.84] | | - | | - | | - | | - | |
| Lignin (g) | AE | 0.13[-0.02;0.28] | 0 | | 0.87[0.72;1.02] | | - | | - | | - | | - | |
| EA Sol.NCP (g) | AE | 0.21[0.09;0.34] | 0 | | 0.79[0.66;0.91] | | - | | - | | - | | - | |
| EA Insol.NCP (g) | CE | 0 | 0.14[0.03;0.25] | | 0.86[0.75;0.97] | | - | | - | | - | | - | |
| EA F22.6cn3.g_adj | ACE | 1.59 x10^-5^[-0.03;0.03] | 7.06 x10^-11^ [-1.65 x10^-5^; 1.65 x10^-5^] | | 1.00[0.97;1.03] | | - | | - | | - | | - | |
| EA F20.5cn3.g_adj | ACE | 1.71 x10^-9^ [-6.5 x10^-5^; 6.5 x10^-5^] | 1.60 x10^-8^ [-1.58 x10^-4^; 1.58 x10^-4^] | | 1.00[1.00;1.00] | | - | | - | | - | | - | |
| EA Riboflavin (mg) | AE | 0.13[0.003;0.25] | | 0 | | 0.87[0.75;0.10] | | - | | - | | - | | - |
| EA AOAC Fibre (g) | AE | 0.29[0.17;0.40] | 0 | | 0.71[0.60;0.83] | | - | | - | | - | | - | |
| EA Selenium (ug) | AE | 0.32[0.21;0.44] | 0 | | 0.68[0.56;0.79] | | - | | - | | - | | - | |

*Moderate to great heritability of nutrients (>20% AE model) estimated using linear structural equation modelling with considering (A) additive genetic effects, (C) environmental effects in common.*
